# Preparation and temperature-controlled morphology of helical microrods composed of supramolecular α-cyclodextrin assemblies†

**DOI:** 10.1039/d3ra07537k

**Published:** 2023-11-23

**Authors:** Toshiyuki Kida, Ayumi Teragaki, Haruya Ishida, Sayaka Sonoda, Momoka Miyasaka, Hajime Shigemitsu

**Affiliations:** a Department of Applied Chemistry, Graduate School of Engineering, Osaka University Suita Japan kida@chem.eng.osaka-u.ac.jp; b Integrated Frontier Research for Medical Science Division, Institute for Open and Transdisciplinary Research Initiatives (OTRI), Osaka University Suita Japan

## Abstract

Significant efforts have been devoted so far to artificially fabricate supramolecular helical nano- and microstructures through the regulated assembly of biological and synthetic building blocks. However, the preparation of supramolecular helical structures with a regulated morphology remains challenging. Here, helical microrods composed of supramolecular α-cyclodextrin (α-CD) assemblies were fabricated by allowing an α-CD/1,1,1,3,3,3-hexafluoro-2-propanol (HFIP)/2-pentanol mixture to stand at 30–60 °C under high humidity conditions. The morphology could be controlled by temperature to produce helical microrods with a regulated pitch and length. These helical rods can be applied as optical devices, chiral separation devices and asymmetric catalysts.

## Abstract 2

Helical microrods comprising supramolecular α-cyclodextrin (α-CD) assemblies are fabricated by allowing an α-CD/HFIP/2-pentanol gel to stand at 30–60 °C under high humidity conditions.

## Graphical abstract

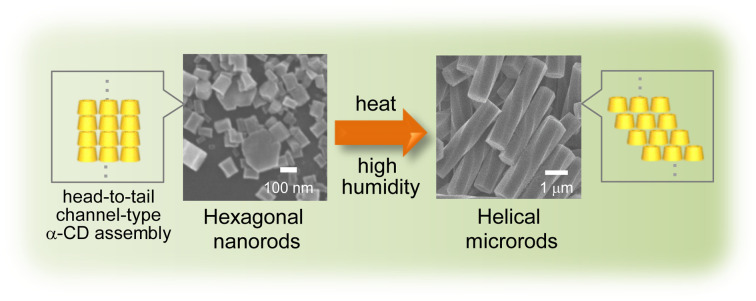

## Introduction

The helical structure is one of the most attractive architectures existing in nature. This structure is widely observed in biomolecules like DNA and proteins.^1^ Significant efforts have been devoted so far to artificially fabricate nanometre- and micrometre-sized supramolecular helical structures through the regulated assembly of biological and synthetic building blocks.^2^ Studies have also been actively conducted to control the morphological characteristics of supramolecular helical structures, such as the handedness, diameter and pitch.^3^ The helical nano- and microstructures thus obtained are expected to have potential applications as functional materials like chiral separation devices,^4^ asymmetric catalysts^2*a*^ and circularly polarised luminescence materials.^5^ However, the preparation of supramolecular helical nano- and microstructures with a regulated morphology remains challenging.

Cyclodextrins (CDs) are a class of cyclic oligosaccharides consisting of several α-(1,4)-linked d-glucopyranose units. They have a hydrophobic cavity inside which a guest molecule with an appropriate size and shape can be included.^6^ CDs can adopt three types of packing structures in crystals, namely, cage-type, channel-type and layer-type structures.^7^ Utilizing the self-assembling ability of CDs, a variety of supramolecular nano- and microstructures have been fabricated.^8^ We previously fabricated cubic microstructures composed of channel-type γ-CD assemblies by mixing an aqueous γ-CD solution with acetone or 2-propanol as a poor solvent, and found that the size of the resulting γ-CD cubes could be easily controlled by the γ-CD concentration, type of poor solvent and inclusion of inorganic ions into the γ-CD cavity.^9^ Recently, we fabricated hexagonal nano- and microplates and microwires composed of channel-type α-CD assembly by mixing a 1,1,1,3,3,3-hexafluoro-2-propanol (HFIP) solution of α-CD with 2-butanol, 2-propanol and 1-propanol as a poor solvent.^10^ These results suggest that the channel-type assembly of CD is a promising building block for the construction of supramolecular nano- and microstructures with a regulated morphology. In the course of our study on the fabrication of nano- and microstructures of α-CD using an α-CD/HFIP solution, we found that supramolecular helical microrods with a uniform size were deposited as white precipitates, which were formed from an organogel comprising a mixture of an α-CD/HFIP solution and 2-pentanol when the organogel was left in a vial for about 10 months at room temperature. This interesting finding prompted us to study the formation mechanism of these helical microrods and to examine the more efficient preparation of helical microrods. Herein, we report the fabrication of α-CD helical microrods with a regulated size and their morphological control by temperature. Although a variety of supramolecular helical polymers have been prepared by the one-dimensional assembly of CD derivatives,^7,11^ there are only a few reports on supramolecular helical nano- and microstructures formed through the three-dimensional assembly of CD building blocks.^12^ In particular, there are no reports on supramolecular helical CD structures with a regulated size. This is the first example of the three-dimensional assembly of CDs to form supramolecular helical microstructures with a regulated length and pitch.

## Experimental section

### General experimental procedures

Scanning Electron Microscope (SEM) measurements were performed with a JSM-6701F instrument (JEOL Ltd., Japan). X-ray diffraction (XRD) patterns of powder samples were obtained at room temperature on a Rigaku RINT InPlane/ultraX18SAXS-IP diffractometer (Rigaku Corporation) using monochromatic Cu Kα radiation generated at 40 kV, 200 mA. The scan rate was 2*θ* = 1° min^−1^ between 2*θ* = 5° and 30°. The oscillatory shear measurements were carried out using a stress-controlled rheometer (HAAKE RheoStress RS 1) with a parallel plate-type geometry (plate diameter 20 mm, plate height 1 mm). The storage modulus G′ and the loss modulus G′′ were measured at a stress of 10 Pa as a function of the angular frequency from 0.1 to 3 rad s^−1^ at 20 °C.

### Preparation of helical rods

α-CD (24 mg) which was dried *in vacuo* at 80 °C for 12 h before use was dissolved in HFIP (1.0 mL) to prepare an α-CD/HFIP solution [25 mM]. This solution (0.50 mL) was added dropwise to 2-pentanol (2.5 mL) stirred at 500 rpm, and the mixture was stirred for 3 h. 0.5 mL of the obtained solution was put into a microtube sealed with parafilm, and was allowed to stand for 3 days to form a gel. After making a hole in the parafilm with a needle (diameter: about 1.1 mm), the microtube was set in a 20 mL vial tube containing 0.5 mL of water. This system was allowed to stand for a prescribed time and temperature (20, 30, 40, 50 and 60 °C). The precipitate formed was separated from the supernatant by filtration, dried with N_2_ flow and subjected to SEM, X-ray diffraction and ^1^H nuclear magnetic resonance (NMR) measurement (solvent: DMSO-*d*_6_). The water content of the precipitate was measured with a HIRANUMA Karl Fischer Titrator AQ-2200 (HIRANUMA Co., Ltd., Japan). The composition of the supernatant was determined by ^1^H NMR measurement and Karl Fischer titration method.

## Results and discussion

**Fig. 1:**
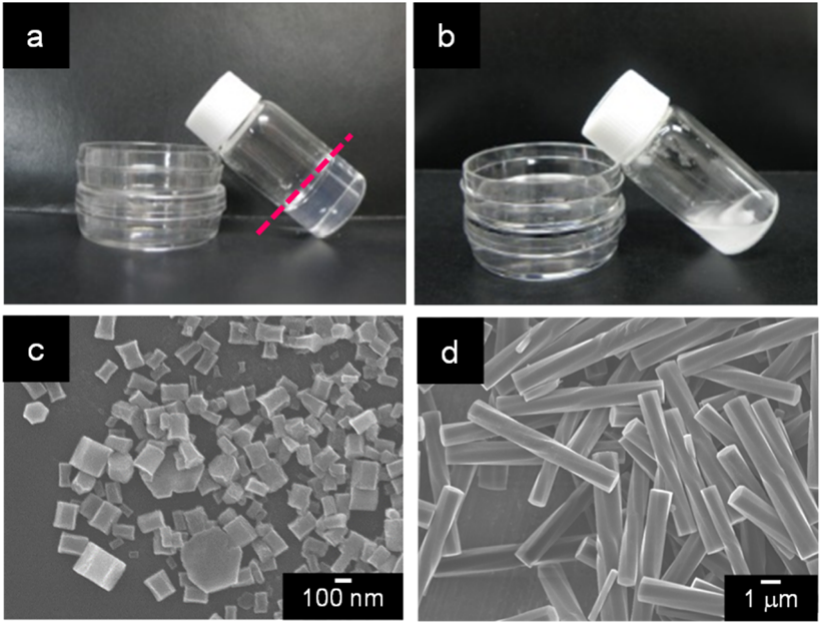
(a, b) Photographs of (a) the gel formed from a mixture of α-CD/HFIP solution (0.5 mL; 25 mM) and 2-pentanol (2.5 mL) after stirring for 3 h, followed by standing for 72 h and (b) a solution containing a white precipitate that was produced from the α-CD/HFIP/2-pentanol gel after about 10 months of storage. SEM images of the supramolecular structures obtained from the (c) gel and (d) precipitate.

**Fig. 2:**
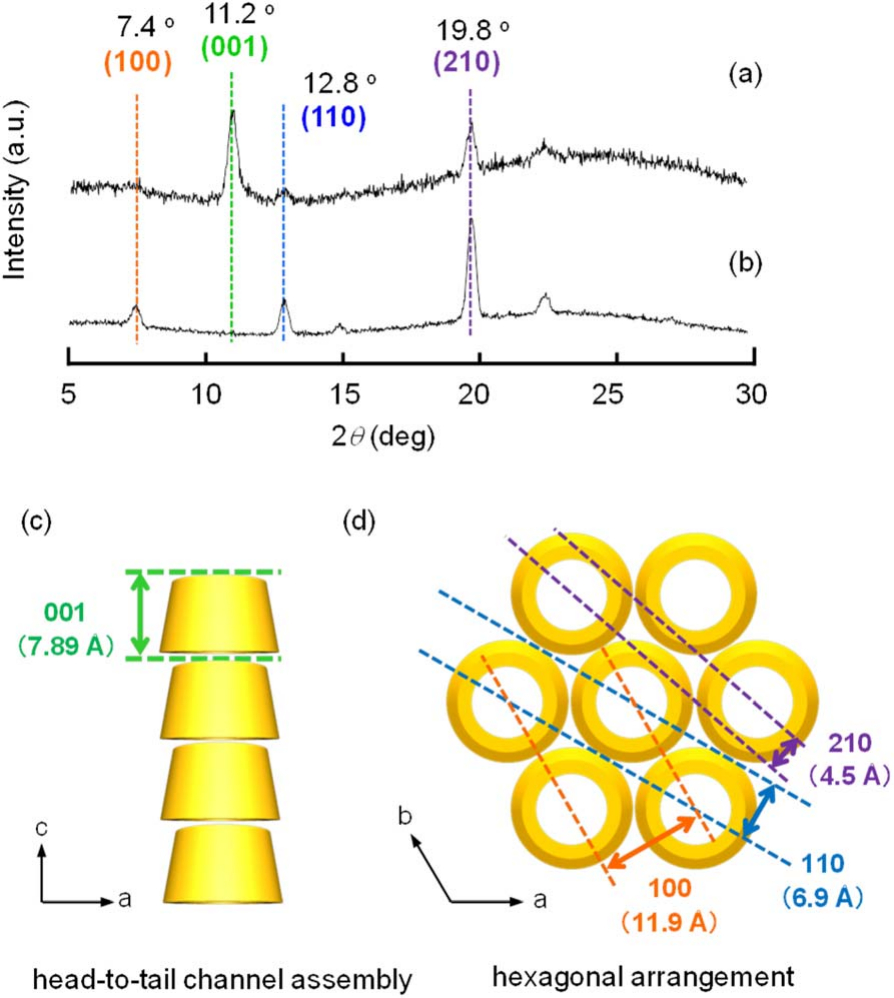
(a, b) XRD patterns of supramolecular structures obtained from the (a) gel and (b) precipitate. Schematic illustration of the diffraction planes of (c) head-to-tail α-CD channel assemblies and (d) hexagonal arrangement of α-CD molecules.

When a mixture of an HFIP solution of α-CD (0.5 mL; 25 mM) and 2-pentanol (2.5 mL) was allowed to stand for 72 h, an organogel that did not exhibit fluidity or fall down upon tilting the vial (Fig. 1a) was obtained. The plots of the storage modulus (G′) and loss modulus (G′′) of this mixture *versus* the angular frequency show that the G′ value is higher than the G′′ value over the angular frequency range of 0.1–3 Hz (Fig. S1†), indicating a permanent network structure. This result confirmed the organogel formation. When this organogel was left in a vial for about 10 months at room temperature, a solution containing a white precipitate was obtained (Fig. 1b). The precipitate was separated from the solution by filtration and dried under N_2_ flow. SEM images of this precipitate showed the formation of left-handed helical hexagonal rods with a uniform size (Fig. 1d). The length (*L*), length of one side of the hexagonal cross section (*r*) and pitch (*P*) of the helical rod were estimated to be 7.75 ± 1.27 μm, 0.52 ± 0.09 μm and 25.53 ± 3.44 μm, respectively. On the other hand, the SEM image of the xerogel obtained by drying the original organogel indicated the formation of non-helical hexagonal rods with sizes of several hundreds of nanometres (Fig. 1c). Thus, the morphological change from non-helical hexagonal nanorods to helical microrods occurred when the α-CD/HFIP/2-pentanol gel was allowed to stand for about 10 months at room temperature. The XRD patterns of the xerogel (hexagonal nanorods) and precipitate (helical microrods) are shown in Fig. 2a and b, respectively. The xerogel showed peaks characteristic of head-to-tail channel assembly of α-CD (2*θ* = 11.2°) (Fig. 2c), with hexagonal alignment along the horizontal direction (2*θ* = 7.4°, 12.8° and 19.8°) (Fig. 2d).^10^ On the other hand, in the XRD patterns of the precipitate, the peaks of the head-to-tail channel assemblies of α-CD disappeared, while the peaks of hexagonally aligned α-CD assembly remained, indicating that the assembly mode of α-CD in the vertical direction changed during the 10 months of storage. ^1^H NMR analysis and water content measurement (by Karl Fischer titration method) of the solution obtained after about 10 months of storage revealed that the water content (2.3 wt%) in the solution was notably higher compared to that in the original gel (0.16 wt%), while the 2-pentanol : HFIP molar ratio was not significantly different from that in the original gel (Fig. S2–S4†). This result suggested that water uptake into the gel during the 10 months of storage changed the assembly mode of α-CD, transforming the morphology of the α-CD structures from hexagonal nanorods to helical microrods.

**Fig. 3:**
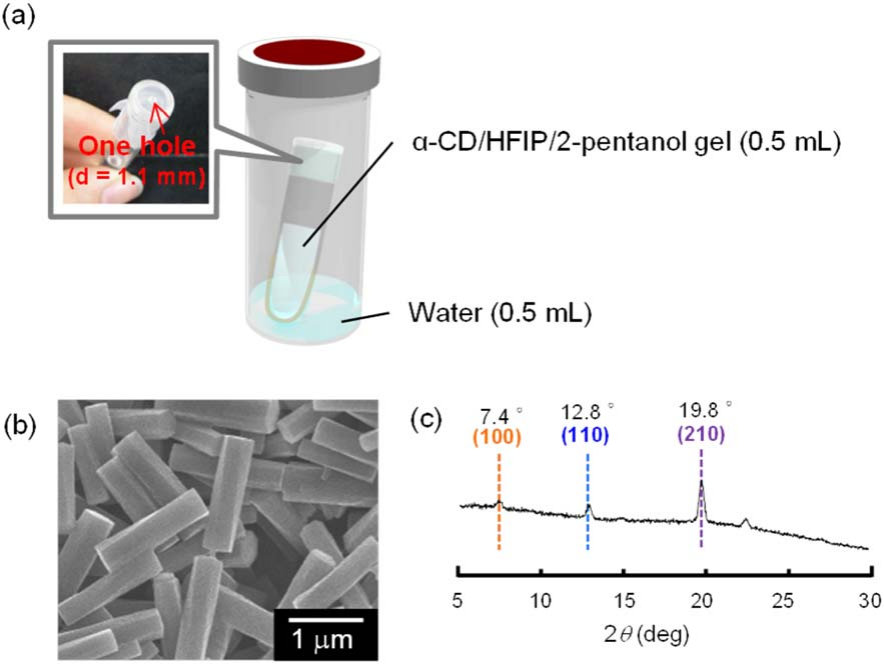
(a) Schematic illustration of a device to fabricate helical rods from the α-CD/HFIP/2-pentanol gel through water uptake into the gel. (b) SEM image and (c) XRD pattern of the precipitate obtained by allowing the α-CD/HFIP/2-pentanol gel to stand for 24 h at 30 °C under high humidity conditions.

Based on this hypothesis, we attempted to fabricate helical microrods from the α-CD/HFIP/2-pentanol gel in a shorter time by controlling the water uptake into the gel. The fabricated experimental device is shown in Fig. 3a. The gel (0.5 mL) was put in a test tube (1 mL) covered with a single perforated parafilm (hole diameter = 1.1 mm). The test tube was placed inside a capped vial (20 mL) containing water (0.5 mL) and kept under high humidity conditions. This device allowed the gradual incorporation of water into the gel.

When the device containing the α-CD/HFIP/2-pentanol gel was heated at 30 °C, a white solid was gradually generated at the surface of the gel. After 24 h, the gel completely collapsed to produce a solution containing a white precipitate. SEM image of the resulting precipitate showed helical microrods with a uniform size (*L* = 1.39 ± 0.16, *r* = 0.18 ± 0.03, *P* = 19.1 ± 4.87 μm; Fig. 3b). XRD pattern of the helical microrods was almost the same as that of the helical microrods obtained by allowing the α-CD/HFIP/2-pentanol gel to stand for about 10 months: there were no peaks characteristic of the head-to-tail channel assembly of α-CD (2*θ* = 11.2°), whereas peaks characteristic of hexagonally packed α-CD assemblies (2*θ* = 7.4°, 12.8° and 19.8°) were observed (Fig. 3c).

**Fig. 4:**
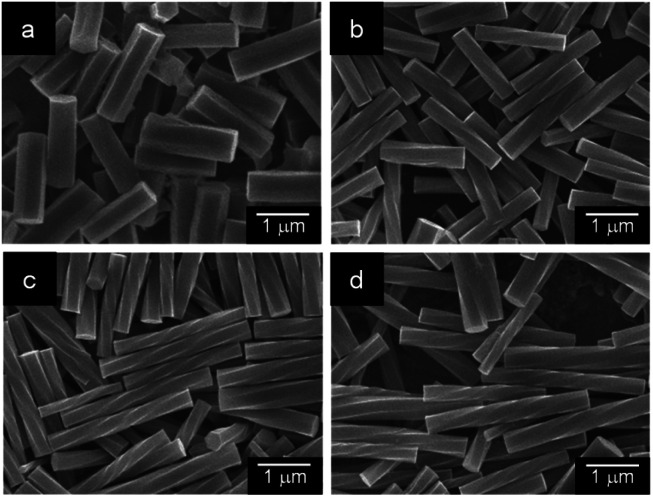
SEM images of the precipitates obtained by allowing the α-CD/HFIP/2-pentanol gel to stand for 24 h under high humidity conditions at (a) 20 °C, (b) 40 °C, (c) 50 °C and (d) 60 °C.

**Fig. 5:**
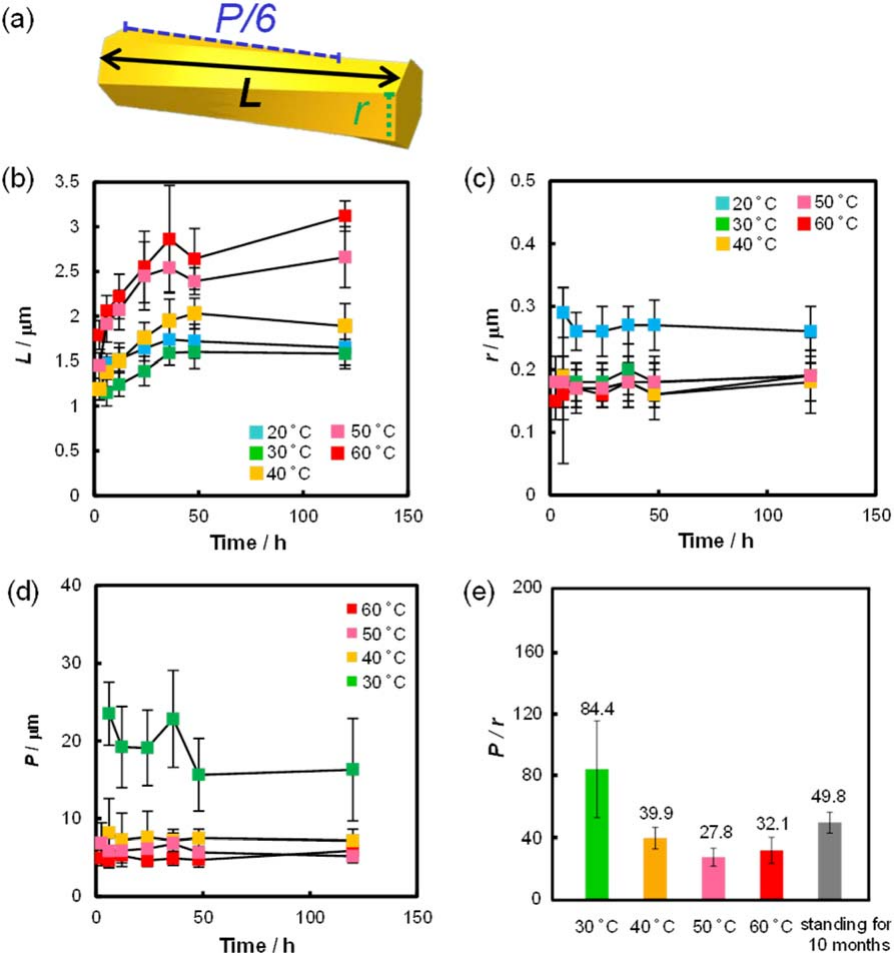
(a) Schematic illustration of the formed helical microrod. *L*, *r* and *P* denote the length, length of one side of the hexagonal cross section and pitch of the helical microrod, respectively. Plots of (b) *L*, (c) *r* and (d) *P* of the helical microrods formed at various standing temperatures *versus* the standing time. (e) *P*/*r* ratio of the helical microrods formed by allowing the gel to stand at various temperatures for 120 h.

Using this device, we examined the effect of temperature and standing time of the gel on the formation of helical rods under high humidity conditions. Fig. 4 shows the SEM images of the precipitates obtained after the gel was left for 24 h at 20, 40, 50 and 60 °C. These images indicated that helical microrods were formed at 40–60 °C, while a mixture of slightly helical and non-helical microrods was formed at 20 °C. Fig. 5b–d shows the plots of *L*, *r* and *P* of the resulting helical microrods *versus* the standing time at different temperatures (Fig. S5–S9†). The length of the helical rod increases as the standing time increases to 36 h. The length also increases with increasing temperature. Fig. 5c indicates that the thickness of the helical rods formed between 30 and 60 °C is not significantly affected by the standing time and temperature. The pitch (*P*) becomes shorter with increasing temperature but is almost unaffected by the standing time. Comparison of the *P*/*r* ratios of the helical rods formed by allowing the gel to stand for 120 h at different temperatures (Fig. 5e) indicates that helical rods with the highest degree of twist were formed at 50 °C.

In the XRD patterns of these rods, only the peaks characteristic of hexagonally packed α-CD assemblies (2*θ* = 7.4°, 12.8° and 19.8°) were observed (Fig. S10–S14†), suggesting that these rods are composed of stacked hexagonally packed α-CD layers.

**Fig. 6:**
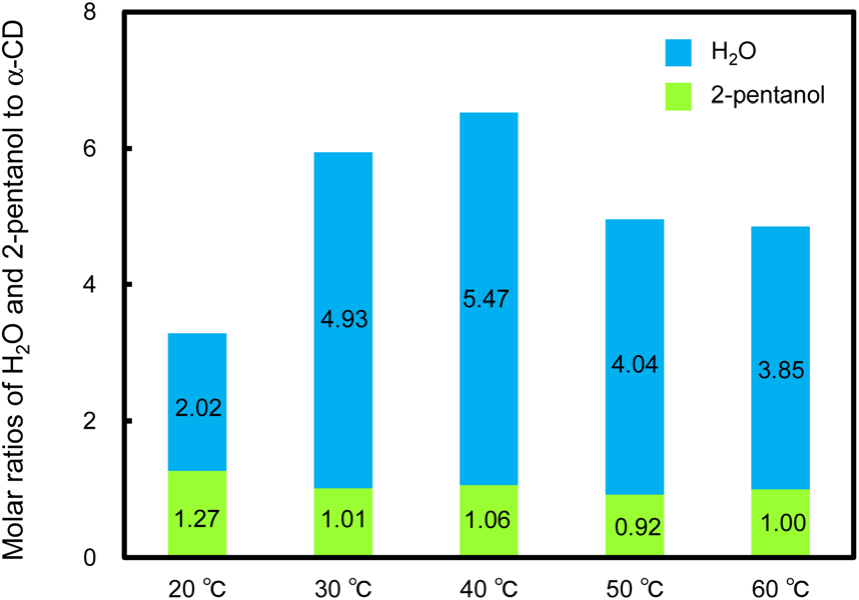
Molar ratios of H_2_O and 2-pentanol to α-CD contained in microrods (helical or non-helical) obtained by allowing the α-CD/HFIP/2-pentanol gel [α-CD/HFIP solution [25 mM] : 2-pentanol = 1 : 5 (v/v)] to stand for 120 h under high humidity conditions at different temperatures (20, 30, 40, 50 and 60 °C).

The molar ratios of 2-pentanol and H_2_O to α-CD in the resulting helical rods were determined by ^1^H NMR analysis and water content measurement (Fig. 6 and S15–S19†). In these helical rods, the 2-pentanol to α-CD molar ratio was approximately 1 : 1 (Fig. 6), regardless of the temperature. Similarly, in the xerogel (hexagonal nanorods) obtained by vacuum-drying of the original α-CD/HFIP/2-pentanol gel, the α-CD and 2-pentanol molar ratio was estimated to be approximately 1 : 1 (Fig. S20†). These results indicate that both the helical microrods and hexagonal nanorods are composed of 1 : 1 inclusion complexes between α-CD and 2-pentanol. The water content increased remarkably with an increase in the standing temperature from 20 to 30 °C and was almost constant from 30 to 60 °C, with slight fluctuations. These results show that water molecules incorporated into the hexagonal nanorods are involved in the morphological change to helical microrods.

**Fig. 7:**
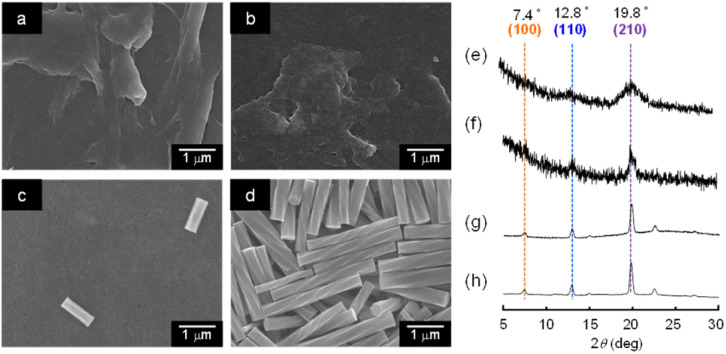
(a–d) SEM images and (e–h) XRD patterns of structures obtained by standing the α-CD/HFIP/2-pentanol gel for (a, e) 30 min, (b, f) 1 h, (c, g) 2.5 h and (d, h) 24 h under high humidity conditions at 50 °C.

To obtain further information about the mechanism of transformation of the hexagonal nanorods to helical microrods, the generation of the helical rods from the α-CD/HFIP/2-pentanol gel was monitored by SEM and XRD. Fig. 7a–d shows the SEM images of the supramolecular structures formed from the gel that was left at 50 °C under high humidity conditions for 30 min, 1 h, 2.5 h and 24 h, respectively. After 30 min, the original hexagonal rods fused together to form a film-like structure. When left for another 2 h, several new rod-like structures with a length of ∼1 μm were generated. These rod-like structures grew over time and transformed into helical rods after 24 h. The XRD patterns of these supramolecular structures are shown in Fig. 7e–h. When the gel was left under high humidity conditions for 30 min, the original peak characteristic of head-to-tail α-CD channel assemblies disappeared, while the peaks of hexagonally packed α-CD assemblies remained, albeit they were broadened. Allowing the gel to stand for a longer time resulted only in sharp peaks characteristic of the hexagonal packing of α-CD molecules, suggesting that the helical rods were formed by the stacking of hexagonally packed α-CD layers. These results suggest that the exposure of the gel to a high humidity environment changes the assembly mode of α-CD (or α-CD-2-pentanol inclusion complexes) through the intervention of water molecules, resulting in the transformation from hexagonal nanorods to helical microrods.

**Fig. 8:**
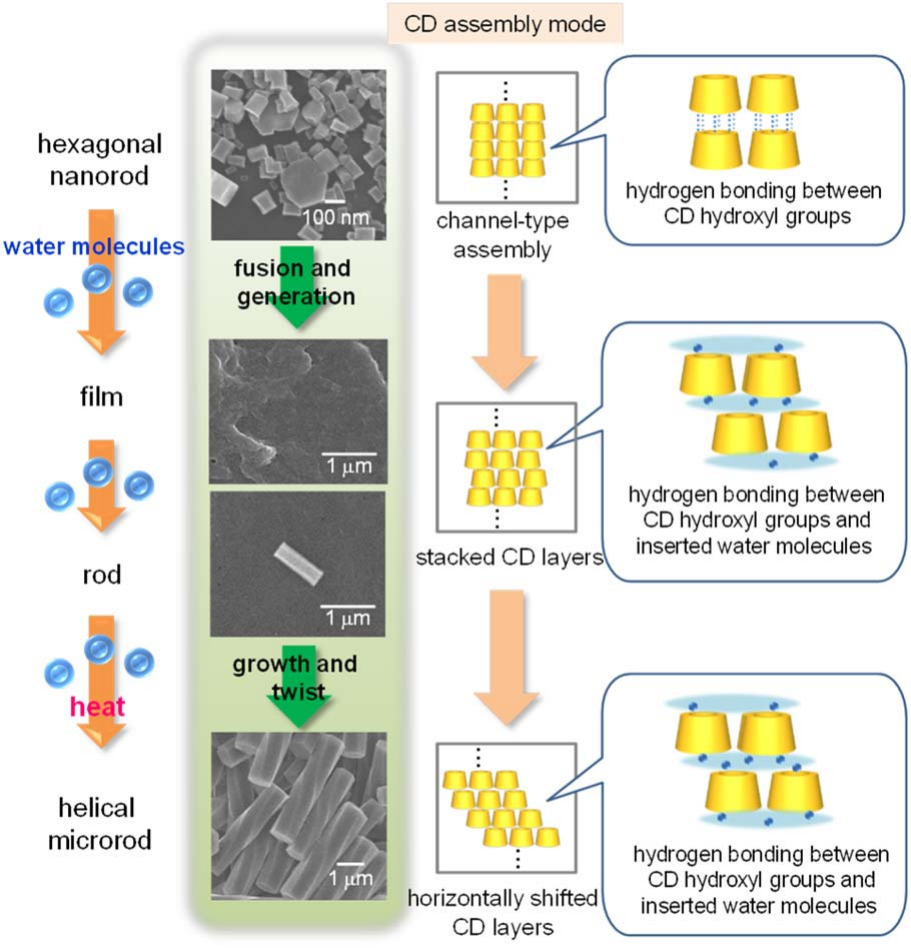
Schematic illustration of a plausible mechanism of helical microrod formation from hexagonal nanorods.

Based on these results, we proposed a plausible mechanism for the formation of helical microrods from hexagonal nanorods (Fig. 8). In the hexagonal nanorods, which constitute the α-CD/HFIP/2-pentanol gel, α-CD molecules form head-to-tail channel-type assemblies that are hexagonally aligned along the horizontal direction, mainly through hydrogen bonds between the hydroxyl groups on the upper and lower rims of α-CD. When the gel is exposed to a high humidity environment, water molecules are incorporated into the α-CD structure, especially between the upper and lower α-CD molecules in the channel assembly. The water uptake induces the aggregation of hexagonal nanorods to form irregular film-like structures, while partially maintaining the hexagonally packed α-CD arrangement. A microrod nucleus composed of hexagonally packed α-CD layers is generated from this film-like structure by continuous uptake of water molecules, following which the α-CD layers grow vertically to form longer microrods owing to hydrogen bonding between the upper and lower α-CD layers through water molecules. Finally, a constant horizontal displacement between the upper and lower α-CD layers caused by heat produces a helical structure of the microrods.

When a mixture of α-CD/HFIP solution and 1-butanol or 2-butanol, which forms an organogel composed of head-to-tail channel-type α-CD assembly^10^ similar to the α-CD/HFIP/2-pentanol mixture, was exposed to a humidity environment under heating, no helical rods were formed. This result suggests that 2-pentanol incorporated into the α-CD cavity also plays an important role in the helical rods formation, probably through stabilization of horizontally shifted α-CD layers.

## Conclusions

We have demonstrated that helical microrods comprising supramolecular α-CD assembly can be easily fabricated by allowing an α-CD/HFIP/2-pentanol gel to stand at 30–60 °C under high humidity conditions. The length and pitch of the helical microrods could be controlled by temperature. Helical rods with the highest degree of twist were formed at 50 °C. These helical rods can be applied as optical devices, chiral separation devices and asymmetric catalysts.

## Author contributions

TK conceived and supervised the project. AT and SS prepared the supramolecular structures. HI, MM and HS analysed the supramolecular structures. TK wrote the manuscript.

## Conflicts of interest

There are no conflicts to declare.

## Supplementary Material

RA-013-D3RA07537K-s001
