# Supplementary material for: Preparation and temperature-controlled morphology of helical microrods composed of supramolecular α-cyclodextrin assemblies
Source: RSC Adv. 2023 Nov 23;13(49):34366–70. doi: 10.1039/d3ra07537k (PMC10665645; doi:10.1039/d3ra07537k)
Supplement: RA-013-D3RA07537K-s001 [file RA-013-D3RA07537K-s001.pdf]

Electronic Supplementary Material (ESI) for RSC Advances.
This journal is © The Royal Society of Chemistry 2023

Supporting Information

Preparation and Temperature-controlled Morphology of Helical Microrods Composed of Supramolecular α-Cyclodextrin Assemblies

Toshiyuki Kida,*[a,b] Ayumi Teragaki,[a] Haruya Ishida,[a] Sayaka Sonoda,[a] Momoka Miyasaka[a] and Hajime Shigemitsu[a,b]

[a]*Department of Applied Chemistry, Graduate School of Engineering, Osaka University, 2-1 Yamada-oka, Suita, Osaka 565-08, Japan*

[b]*Integrated Frontier Research for Medical Science Division, Institute for Open and Transdisciplinary Research Initiatives (OTRI), Osaka University, 2-1 Yamada-oka, Suita, Osaka 565-0871, Japan*

Contents

1. **Rheological properties of organogels formed by mixing a HFIP solution of α-CD and 2-pentanol**
2. **2-Pentanol/HFIP molar ratio in the α-CD/HFIP/2-pentanol gel and the solution (supernatant) produced from the gel after about 10 months of storage**
3. **SEM images of precipitates obtained by allowing the α-CD/HFIP/2-pentanol gel to stand under high humidity conditions**
4. **XRD patterns of precipitates obtained by allowing the α-CD/HFIP/2-pentanol gel to stand under high humidity conditions**
5. **Molar ratios of H_2O and 2-pentanol to α-CD contained in microrods**

1. **Rheological properties of organogels formed by mixing a HFIP solution of α-CD and 2-pentanol**

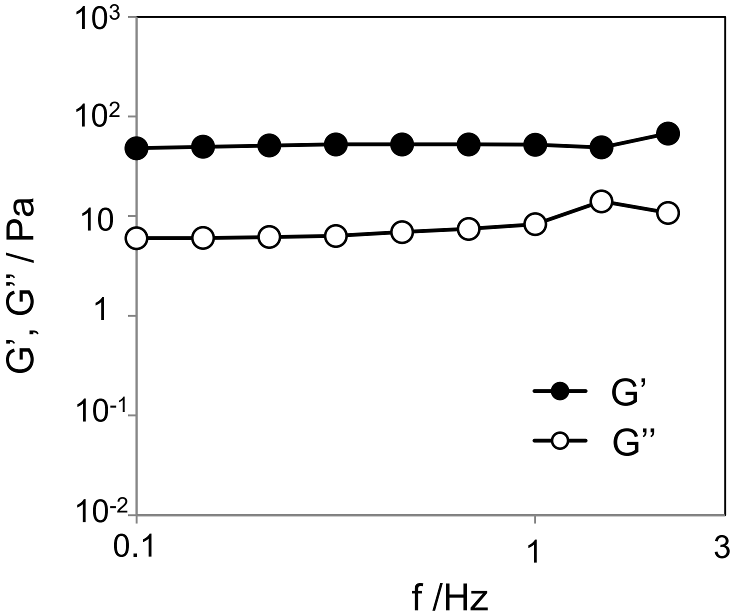

Figure S1. Plots of the storage modulus (G') and the loss modulus (G'') of organogels, which were formed by stirring a mixture of an α-CD/HFIP solution and 2-pentanol for 3 h, followed by allowing to stand for 72 h, against angular frequency (stress: 10 Pa).

2. **2-Pentanol/HFIP molar ratio in the α-CD/HFIP/2-pentanol gel and the solution (supernatant) produced from the gel after about 10 months of storage**

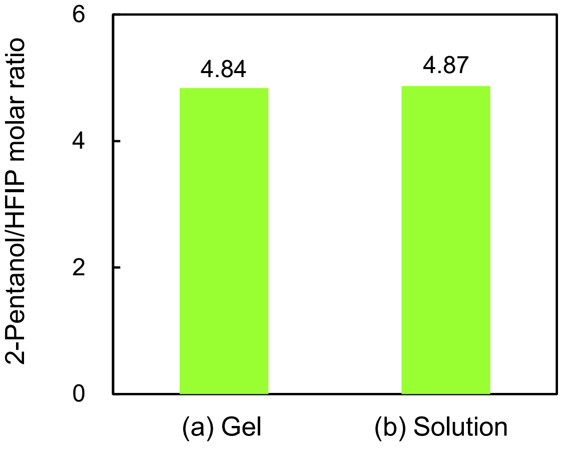

Figure S2. Molar ratio of 2-pentanol to HFIP (a) in the gel formed by stirring a mixture of α-CD/HFIP solution (0.5 mL) [25 mM] and 2-pentanol (2.5 mL) for 3 h, followed by standing for 72 h, and (b) in the solution (supernatant) produced from the gel after standing for about 10 months.

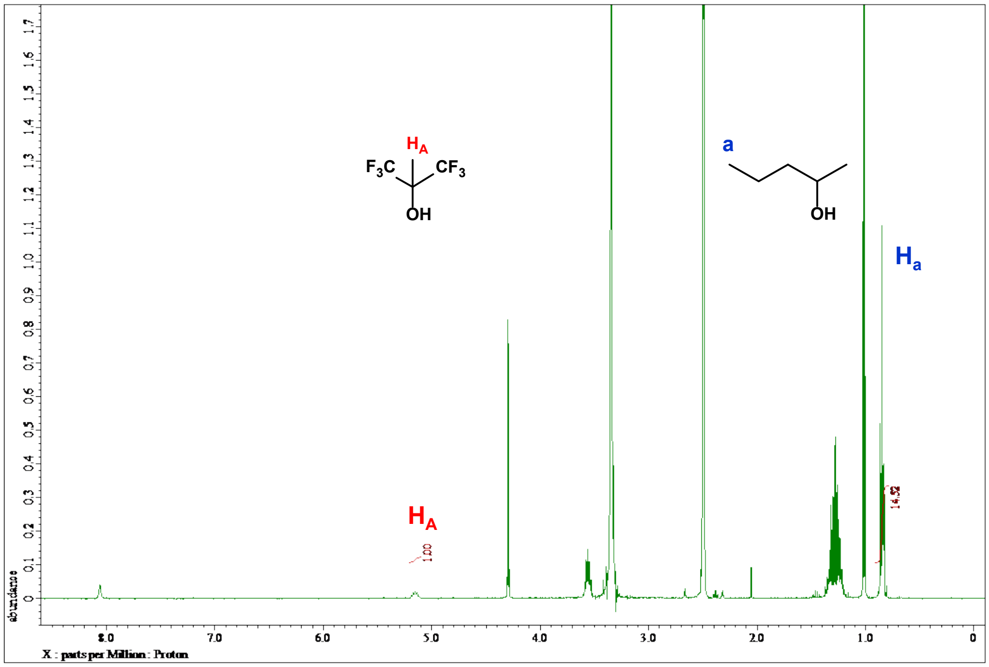

Figure S3. ^{1}H NMR spectrum (solvent: DMSO-d_6) of the gel formed by stirring a mixture of α-CD/HFIP solution (0.5 mL) [25 mM] and 2-pentanol (2.5 mL) for 3 h, followed by standing for 72 h.

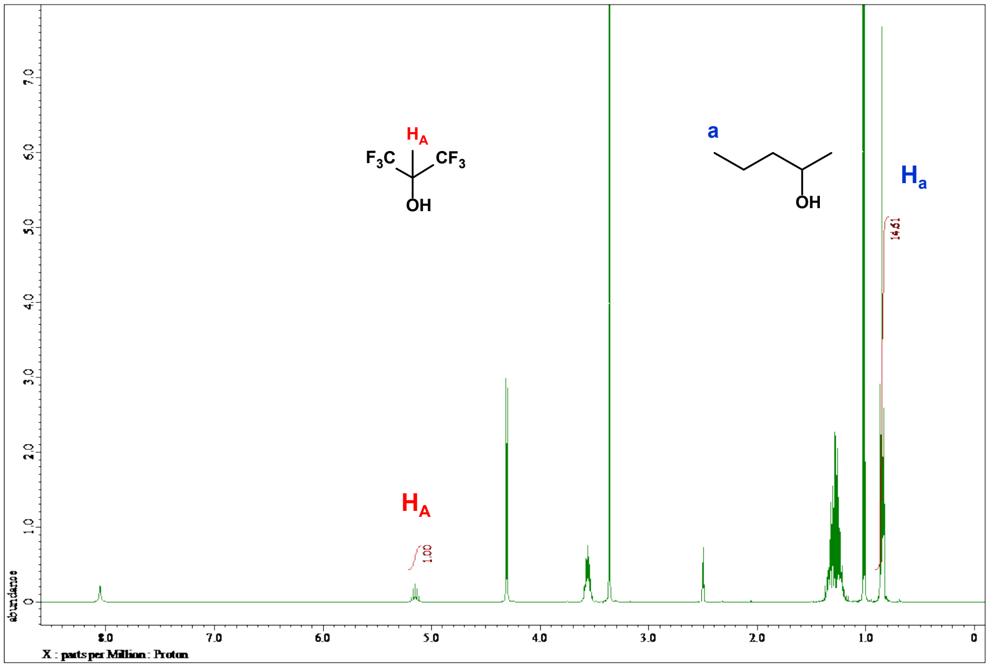

Figure S4. ^{1}H NMR spectrum (solvent: DMSO-d_6) of the solution (supernatant) produced from the α-CD/HFIP/2-pentanol gel after standing for about 10 months.

3. **SEM images of precipitates obtained by allowing the α-CD/HFIP/2-pentanol gel to stand under high humidity conditions**

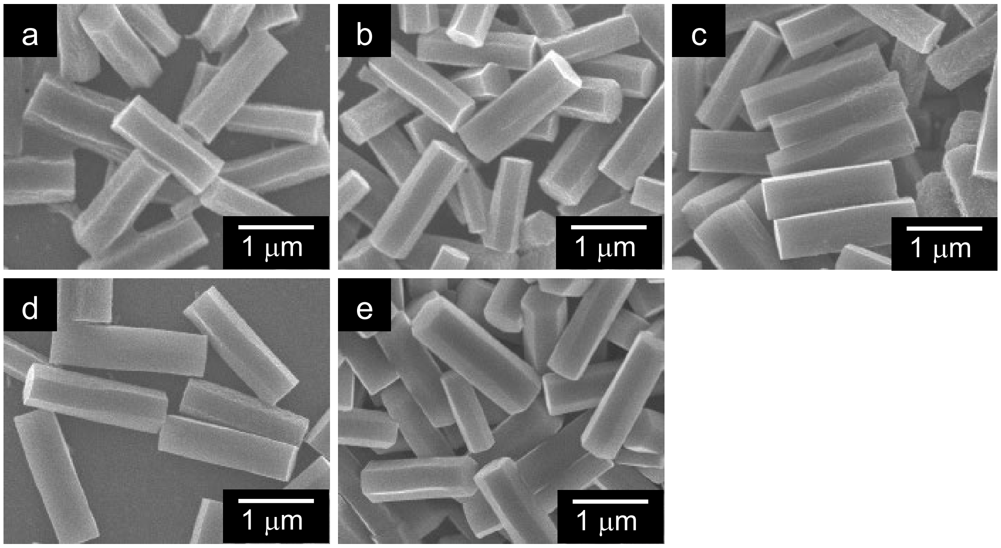

Figure S5. SEM images of precipitates obtained by allowing the α-CD/HFIP/2-pentanol gel [an α-CD/HFIP solution [25 mM]:2-pentanol = 1:5 (v/v)] to stand at 20 °C under high humidity conditions for (a) 6 h, (b) 12 h, (c) 36 h, (d) 48 h and (e) 120 h.

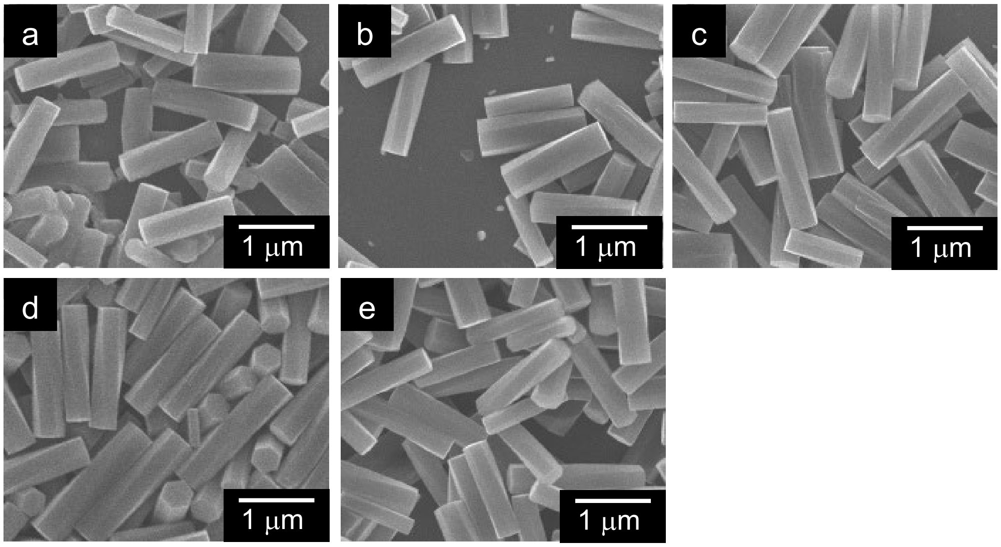

Figure S6. SEM images of precipitates obtained by allowing the α-CD/HFIP/2-pentanol gel [an α-CD/HFIP solution [25 mM]:2-pentanol = 1:5 (v/v)] to stand at 30 °C under high humidity conditions for (a) 6 h, (b) 12 h, (c) 36 h, (d) 48 h and (e) 120 h.

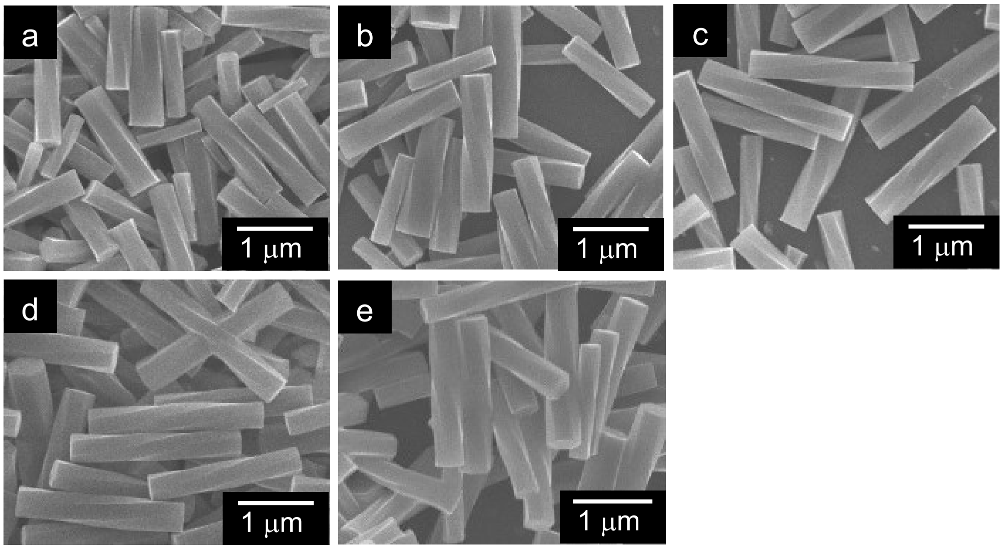

Figure S7. SEM images of precipitates obtained by allowing the α-CD/HFIP/2-pentanol gel [an α-CD/HFIP solution [25 mM]:2-pentanol = 1:5 (v/v)] to stand at 40 °C under high humidity conditions for (a) 6 h, (b) 12 h, (c) 36 h, (d) 48 h and (e) 120 h.

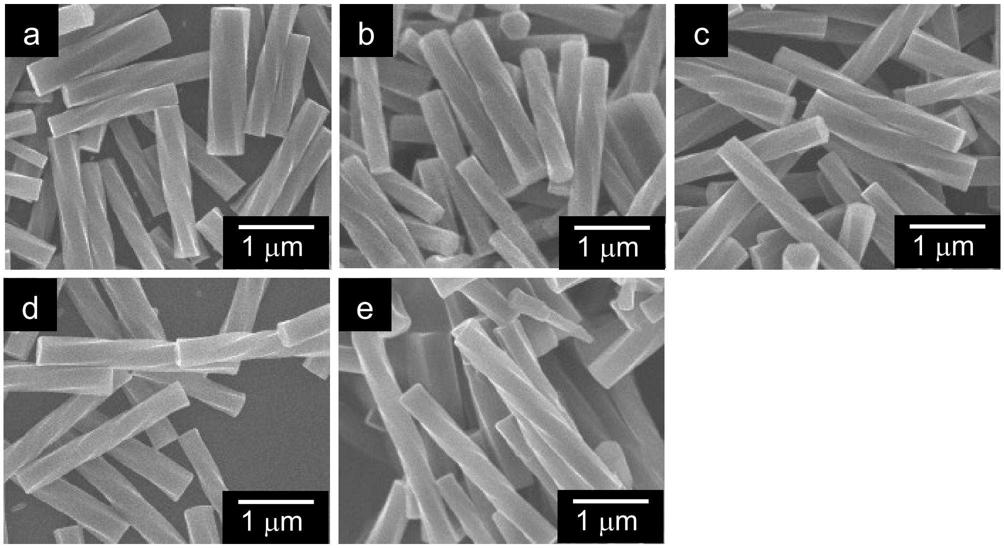

Figure S8. SEM images of precipitates obtained by allowing the α-CD/HFIP/2-pentanol gel [an α-CD/HFIP solution [25 mM]:2-pentanol = 1:5 (v/v)] to stand at 50 °C under high humidity conditions for (a) 6 h, (b) 12 h, (c) 36 h, (d) 48 h and (e) 120 h.

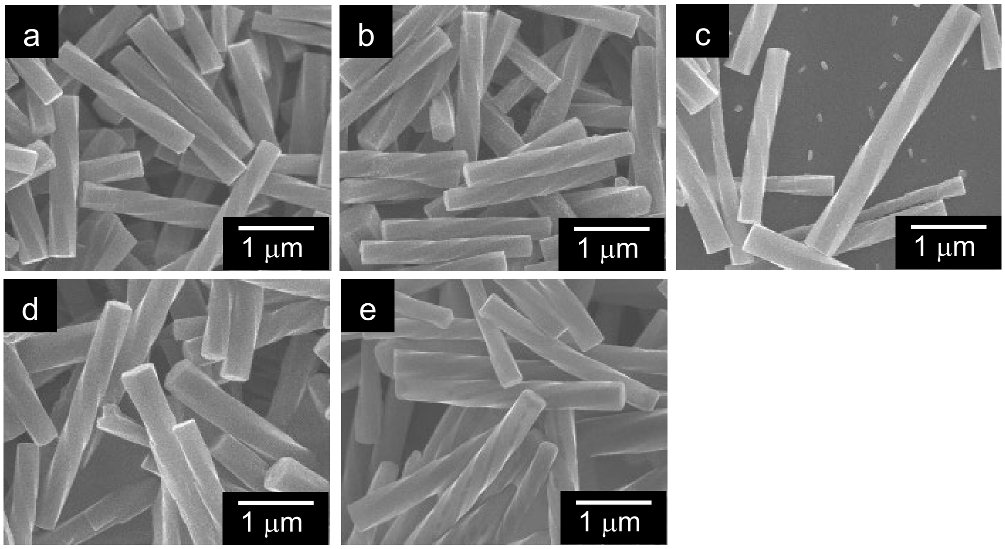

Figure S9. SEM images of precipitates obtained by allowing the α-CD/HFIP/2-pentanol gel [an α-CD/HFIP solution [25 mM]:2-pentanol = 1:5 (v/v)] to stand at 60 °C under high humidity conditions for (a) 6 h, (b) 12 h, (c) 36 h, (d) 48 h and (e) 120 h.

4. XRD patterns of precipitates obtained by allowing the α-CD/HFIP/2-pentanol gel to stand under high humidity conditions

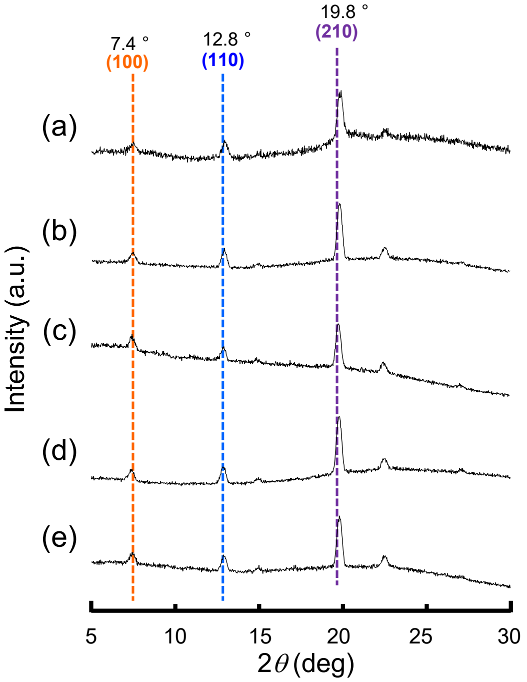

Figure S10. XRD patterns of precipitates obtained by allowing the α-CD/HFIP/2-pentanol gel [an α-CD/HFIP solution [25 mM]:2-pentanol = 1:5 (v/v)] to stand at 20 °C under high humidity conditions for (a) 6 h, (b) 12 h, (c) 36 h, (d) 48 h and (e) 120 h.

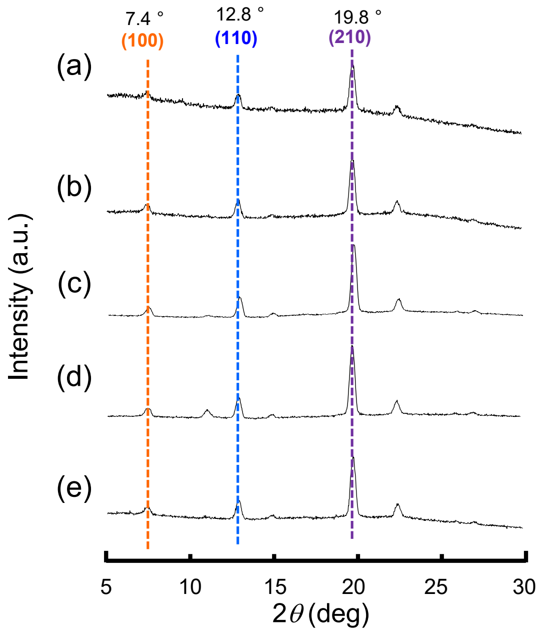

Figure S11. XRD patterns of precipitates obtained by allowing the α-CD/HFIP/2-pentanol gel [an α-CD/HFIP solution [25 mM]:2-pentanol = 1:5 (v/v)] to stand at 30 °C under high humidity conditions for (a) 6 h, (b) 12 h, (c) 36 h, (d) 48 h and (e) 120 h.

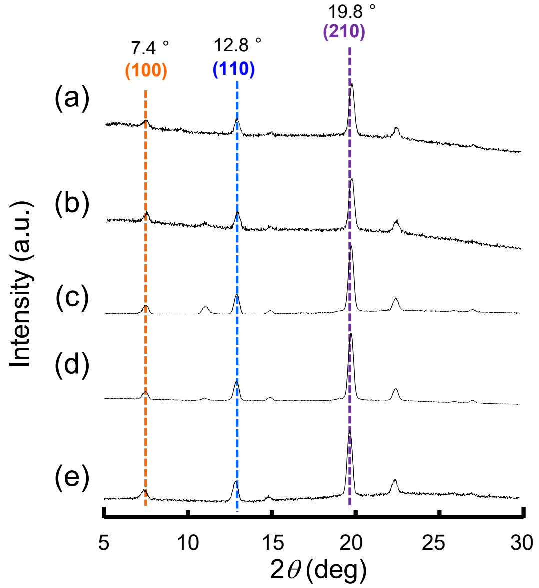

Figure S12. XRD patterns of precipitates obtained by allowing the α-CD/HFIP/2-pentanol gel [an α-CD/HFIP solution [25 mM]:2-pentanol = 1:5 (v/v)] to stand at 40 °C under high humidity conditions for (a) 6 h, (b) 12 h, (c) 36 h, (d) 48 h and (e) 120 h.

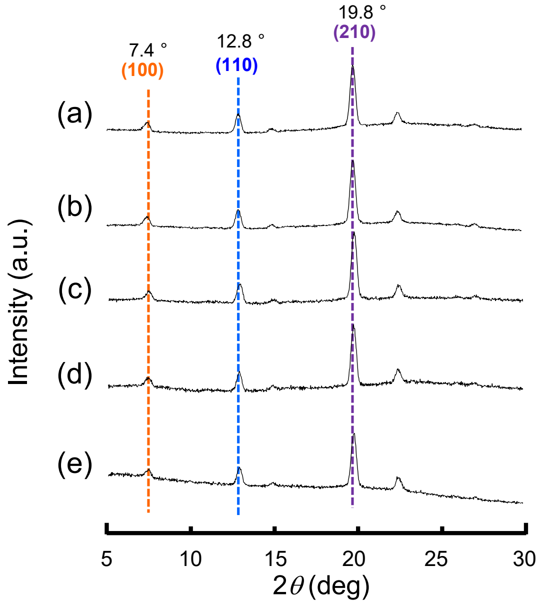

Figure S13. XRD patterns of precipitates obtained by allowing the α-CD/HFIP/2-pentanol gel [an α-CD/HFIP solution [25 mM]:2-pentanol = 1:5 (v/v)] to stand at 50 °C under high humidity conditions for (a) 6 h, (b) 12 h, (c) 36 h, (d) 48 h and (e) 120 h.

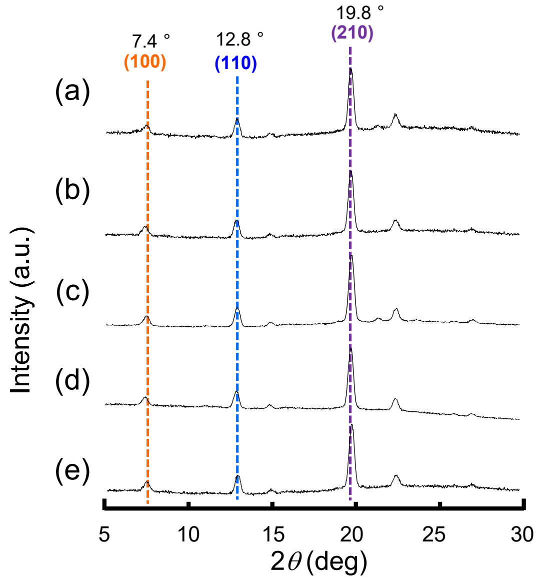

Figure S14. XRD patterns of precipitates obtained by allowing the α-CD/HFIP/2-pentanol gel [an α-CD/HFIP solution [25 mM]:2-pentanol = 1:5 (v/v)] to stand at 60 °C under high humidity conditions for (a) 6 h, (b) 12 h, (c) 36 h, (d) 48 h and (e) 120 h.

5. Molar ratios of H_2O and 2-pentanol to α-CD contained in microrods

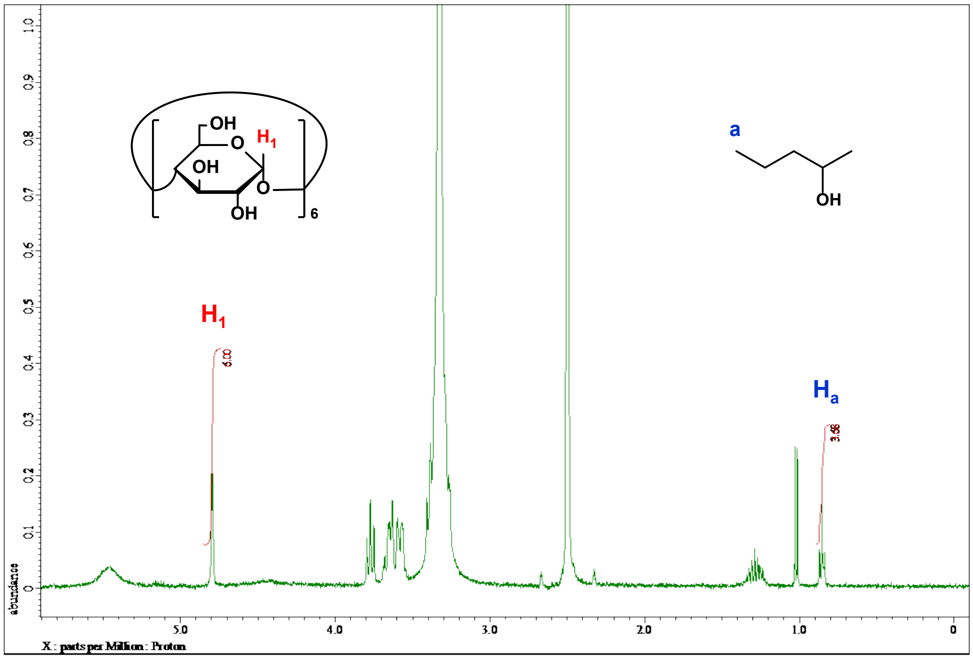

Figure S15. ^{1}H NMR spectrum (solvent: DMSO-d_6) of microrods obtained by allowing the α-CD/HFIP/2-pentanol gel to stand for 120 h at 20 °C under high humidity conditions.

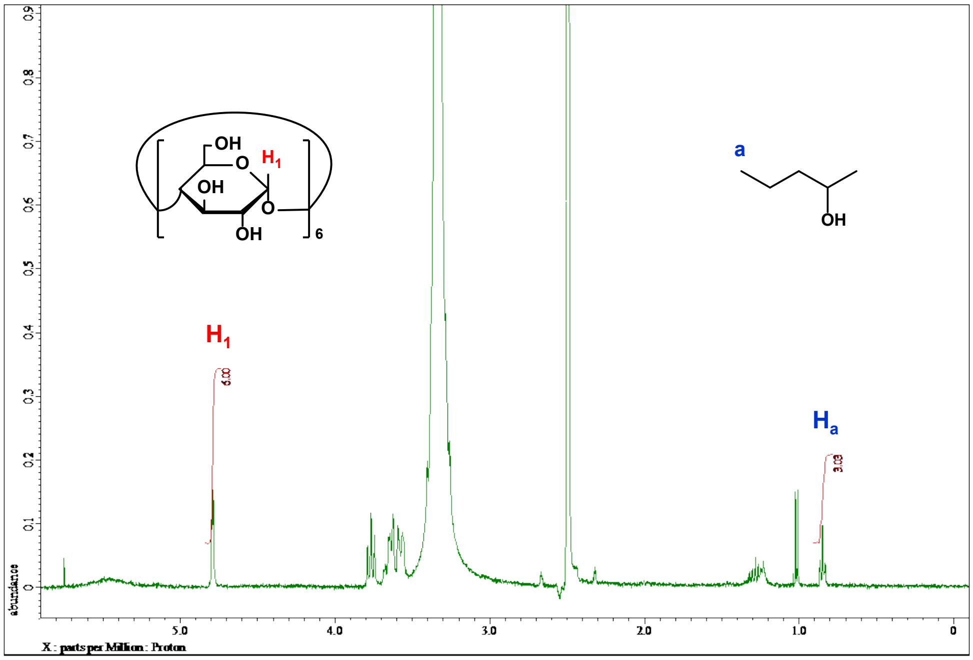

Figure S16. ^{1}H NMR spectrum (solvent: DMSO-d_6) of microrods obtained by allowing the α-CD/HFIP/2-pentanol gel to stand for 120 h at 30 °C under high humidity conditions.

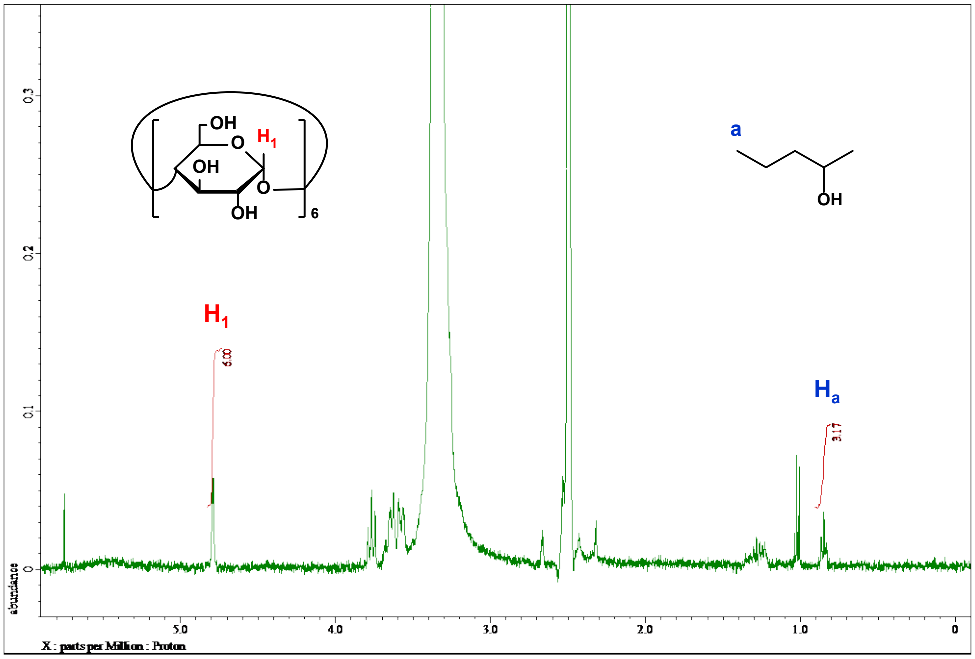

Figure S17. ^{1}H NMR spectrum (solvent: DMSO-d_6) of microrods obtained by allowing the α-CD/HFIP/2-pentanol gel to stand for 120 h at 40 °C under high humidity conditions.

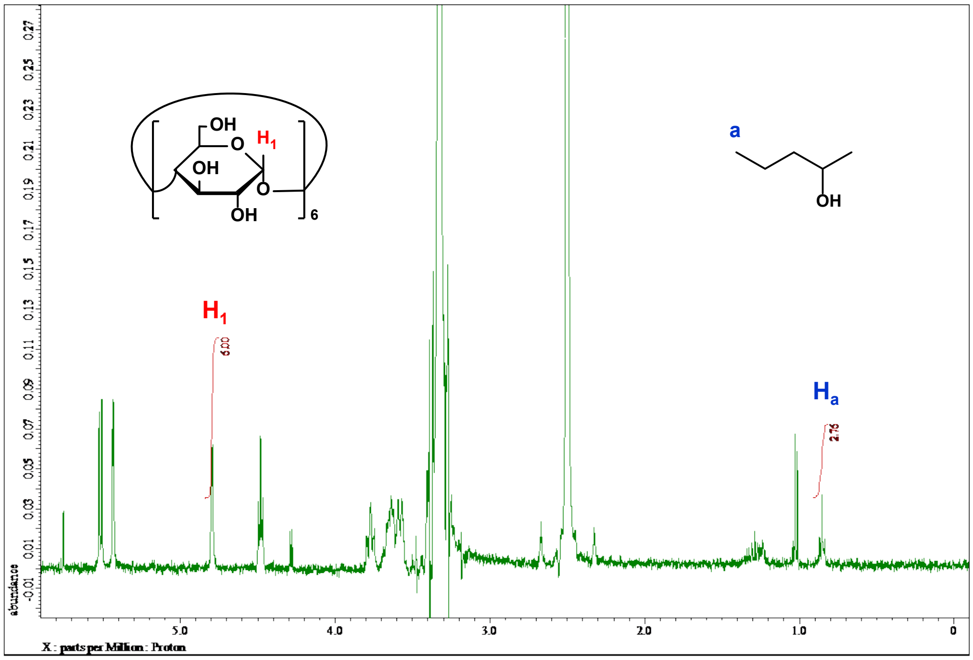

Figure S18. ^{1}H NMR spectrum (solvent: DMSO-d_6) of microrods obtained by allowing the α-CD/HFIP/2-pentanol gel to stand for 120 h at 50 °C under high humidity conditions.

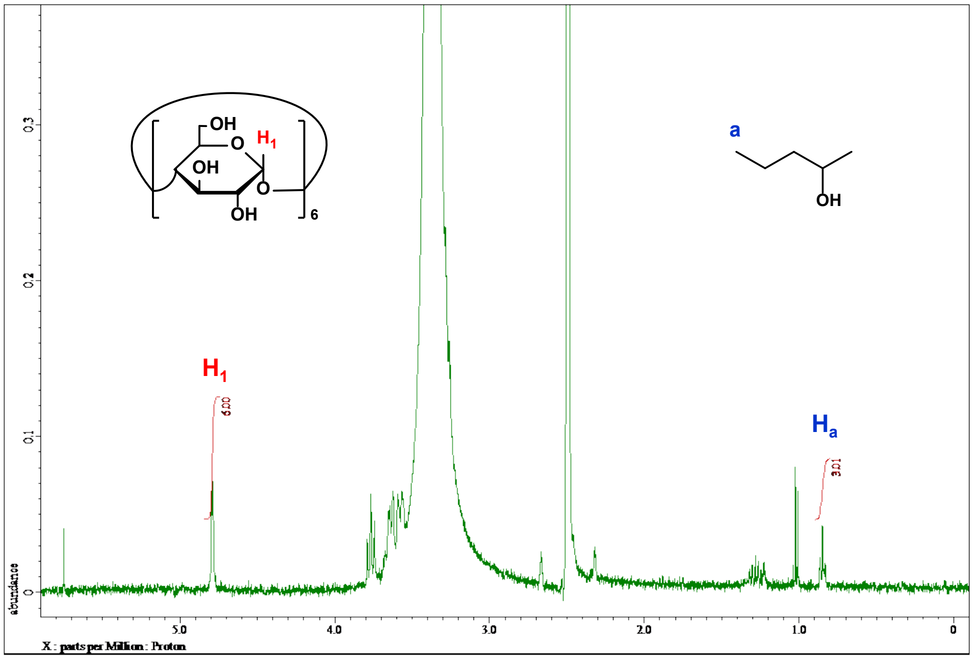

Figure S19. ^{1}H NMR spectrum (solvent: DMSO-d_6) of microrods obtained by allowing the α-CD/HFIP/2-pentanol gel to stand for 120 h at 60 °C under high humidity conditions.

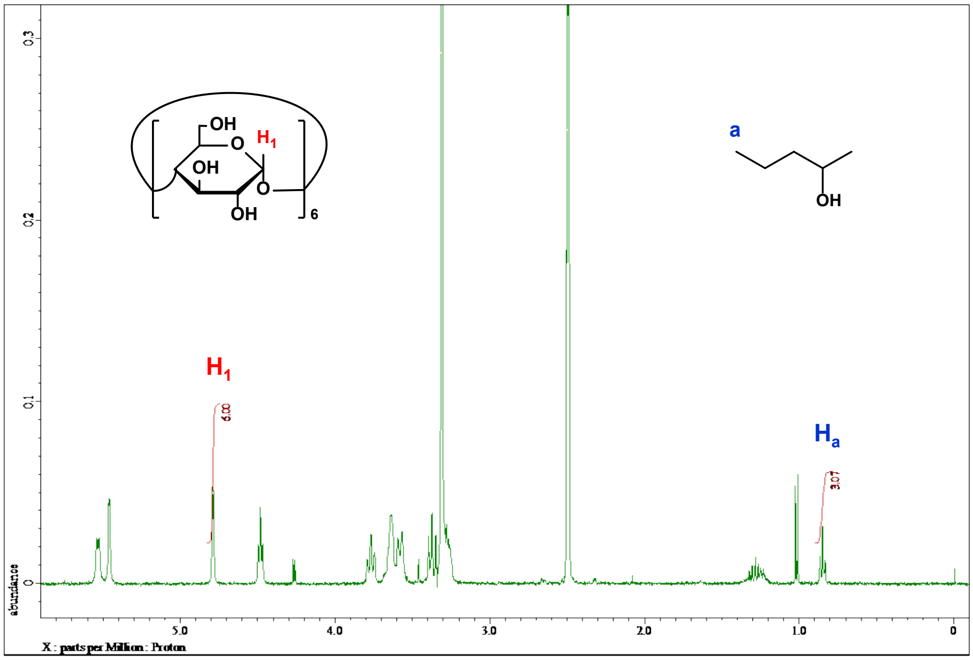

Figure S20. ^{1}H NMR spectrum (solvent: DMSO-d_6) of the solid obtained after drying the α-CD/HFIP/2-pentanol gel for 24 h *in vacuo*.
